# Influence of Binder Reactivity and Grain Size Fraction on the Technological, Mechanical, and Thermophysical Properties of Core Moulding Sands

**DOI:** 10.3390/ma19020361

**Published:** 2026-01-16

**Authors:** Grzegorz Piwowarski, Faustyna Woźniak, Artur Bobrowski

**Affiliations:** Faculty of Foundry Engineering, AGH University of Krakow, Reymonta 23, 30-059 Krakow, Poland; wozfau@agh.edu.pl (F.W.); arturb@agh.edu.pl (A.B.)

**Keywords:** phenolic resole binder, binder reactivity, core sands, quartz sand granulometry, thermophysical properties, heat accumulation, thermal conductivity

## Abstract

The properties of chemically bonded core sands strongly depend on the reactivity of phenol-formaldehyde resole binders and on the granulometry of the sand matrix. This study presents an evaluation of the mechanical, technological, thermomechanical, and thermophysical properties of core sands prepared using two resole binders with different reactivity levels (Resin 1—lower reactivity; Resin 2—higher reactivity) and two fractions of quartz sand (BK 40 and BK 45). The investigations included the kinetics of strength development (1–48 h), friability, permeability, thermal deformation (DMA), and the determination of thermophysical coefficients (*λ*_2_, *a*_2_, *b*_2_) based on temperature field registration during the solidification of a copper plate. The results indicate that sands containing the higher-reactivity binder exhibit a faster early strength increase (≈0.42–0.45 MPa after 1–3 h), whereas sands bonded with the lower-reactivity resin reach higher tensile strength after 24–48 h (≈0.58–0.62 MPa). Specimens based on BK 45 quartz sand achieved higher tensile strength; however, the finer grain fraction resulted in increased friability (up to ≈3.97%) and a reduction in permeability by 30–40%. DMA analysis confirmed that sands based on BK 40 exhibit delayed and more stable thermal deformation. Thermophysical parameters revealed that BK 45 provides significantly higher thermal insulation, extending the solidification time of the Cu plate from 71–73 s to 89–92 s compared with BK 40. Overall, the results indicate that the combination of BK 40 quartz sand and a lower-reactivity resin offers an optimal balance between thermal conductivity and thermal stability, promoting improved technological performance in casting processes. The determined thermophysical coefficients can be directly applied as input data for foundry process simulations.

## 1. Introduction

Modern foundry practice requires precise control of mould and core sand properties, as their composition, rheology, and performance directly determine process stability and casting quality [1,2,3]. Among the factors governing the behaviour of core sands, the type of binder and the granulometry of the sand matrix play a dominant role, jointly influencing the formation of polymer bridges, curing kinetics, porosity, and gas flow within the core. Sand granulometry, specific surface area, and grain-size uniformity determine the packing density of grains and, consequently, the strength, friability, and gas evolution characteristics of cores and moulds [4,5,6].

Phenol-formaldehyde resole resins remain one of the most important groups of organic binders used in no-bake and alkaline phenolic no-bake (APNB) technologies due to their favourable rheological properties, adjustable reactivity, and widespread industrial applicability [7,8,9]. Binder reactivity directly affects the kinetics of polycondensation and the formation of intergranular bonding bridges, thereby determining the mechanical properties of cores, including cold compressive strength and structural resistance under high-temperature conditions [10,11].

These relationships have been confirmed in previous studies on cores produced with resins of different reactivity levels and subjected to varying environmental conditions [12]. One of the key challenges in core sand technology is binder degradation occurring during storage under elevated relative humidity, ambient temperature, and prolonged storage time. Studies have shown that phenol-formaldehyde resole binders are susceptible to moisture sorption, surface restructuring, and local hydrolysis, leading to a reduction in the number of bonding bridges and weakening of the core structure [11,12,13,14,15,16]. As a result, technological properties such as resistance to erosion by molten metal, gas evacuation capability, and mechanical strength after storage deteriorate markedly. Moreover, increased binder moisture content may intensify gas evolution during pouring, adversely affecting casting quality [17].

The sand matrix additionally influences core behaviour under thermal loading. Grain size and packing structure affect thermal conductivity, heating rate, and the intensity of thermal degradation of resin bonds (polymer bridges between sand grains) upon contact with molten metal. Numerous studies have demonstrated that the thermal and thermomechanical properties of cores determine their dimensional stability, resistance to deformation, and the duration for which structural integrity is maintained [7,14,15,16]. In light of these considerations, a comprehensive approach is required, encompassing both the evaluation of mechanical and technological properties and the investigation of core behaviour under thermal loading and degradation mechanisms related to sand composition.

The type of sand matrix and binder affects not only the technological and mechanical properties of moulds and cores but also their thermophysical properties. Foundry moulds and cores must exhibit sufficient strength to preserve the desired casting geometry, while their thermophysical parameters govern the rate of heat extraction from the solidifying and cooling casting. These parameters significantly influence the internal structure of the casting and its subsequent mechanical properties [18,19]. Various methods are available for determining the basic thermophysical coefficients characterising foundry materials. Most of these techniques provide data at a single temperature or within a limited temperature range, such as Laser Flash Analysis (LFA), the Hot Disk method, or radial heat flow techniques [20,21,22,23].

However, despite extensive research on the mechanical and technological properties of core sands, the combined influence of binder reactivity and sand granulometry on temperature-dependent thermophysical properties determined under real casting conditions has not been sufficiently addressed in the literature. However, to capture the actual evolution of thermophysical properties during pouring and cooling, data must be collected under real process conditions. This is achieved by registering the temperature distribution within the mould during casting solidification and cooling [24,25,26,27]. The recorded temperature field during the initial solidification period enables the determination of fundamental thermophysical coefficients of mould and core materials, including thermal conductivity *λ*_2_, thermal diffusivity *a*_2_, and heat accumulation coefficient *b*_2_. These coefficients depend on mould compaction, sand granulometry, and binder type [28] and vary significantly with temperature. Thermophysical coefficients describe how rapidly a mould material extracts heat from the casting, how much heat it can absorb over time, and how much thermal energy it can store. Proper selection of mould and core materials therefore has a substantial impact on casting microstructure and properties, beyond the intrinsic characteristics of the alloy. Accurate thermophysical data allow for the selection of appropriate materials for specific technologies and are essential for reliable numerical simulations, which are increasingly used to optimize foundry processes. To ensure simulation accuracy, input parameters must reflect the real properties of the materials employed, underscoring the necessity of determining temperature-dependent thermophysical coefficients.

The objective of this study was to analyse the influence of phenol-formaldehyde resole binders with different reactivity levels and sand matrix granulometries on the thermophysical and technological properties of core sands. Four binder-sand combinations were examined, for which thermal conductivity (*λ*_2_), thermal diffusivity (*a*_2_), and heat accumulation coefficient (*b*_2_) were determined as functions of temperature. Additionally, the effect of these parameters on metal solidification time and core structural integrity after pouring was assessed. The obtained results provide valuable input data for numerical modelling of casting processes and support the design of mould materials with controlled thermal conductivity and dimensional stability. The novelty of the present work lies in the simultaneous evaluation of mechanical, thermomechanical, and thermophysical properties of core sands combined with temperature-field-based determination of thermophysical coefficients under real solidification conditions.

## 2. Materials and Methods

### 2.1. Materials

Two alkaline phenol-formaldehyde resole-type resins differing in their degree of reactivity were used in the study, together with a hardener belonging to the group of dicarboxylic acid esters. Quartz sand with two grain size fractions—BK 40 and BK 45—was employed as the base material for the preparation of the core sand mixtures, enabling an assessment of the effect of sand granulometry on the properties of the resulting cores. The basic physicochemical properties of the individual resins are summarized in Table 1.

The selection of materials and mixture proportions was based on their widespread industrial application in no-bake and APNB core technologies, as well as on previous studies demonstrating the sensitivity of phenol-formaldehyde resole binders to changes in reactivity and sand granulometry [4,7,12,17]. The resin content (1.5 wt.% relative to sand) and hardener dosage (25 wt.% relative to resin) correspond to typical industrial practice and were selected to ensure sufficient strength development while maintaining acceptable permeability and friability levels. The use of two quartz sand fractions (BK 40 and BK 45) with narrow grain size distributions enabled isolation of the granulometric effect on mechanical, technological, and thermophysical properties.

### 2.2. Methods

#### 2.2.1. Technological and Mechanical Properties

**Figure 1:**
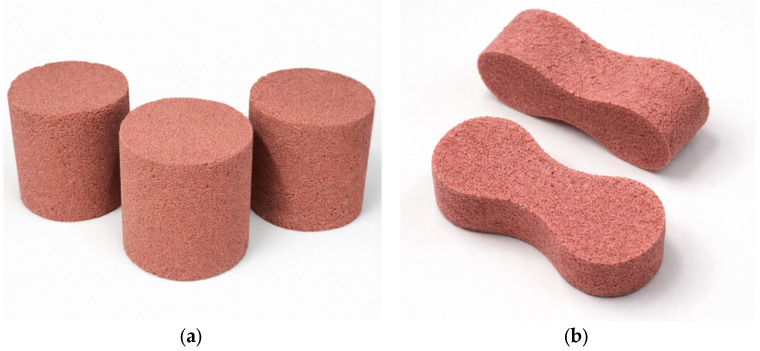
Representative core specimens used in the study: (**a**) cylindrical samples with dimensions Ø50 × 50 mm, and (**b**) dog-bone-shaped specimens prepared for tensile strength testing.

As part of the study, cores were produced in the form of cylindrical specimens with dimensions of Ø50 × 50 mm as well as dog-bone-shaped specimens intended for tensile strength testing. The cores were prepared using the Alpha-Set technology, classified as a self-setting (no-bake) process (Figure 1).

The dog-bone specimens were formed by vibrational compaction in standardized moulds. These specimens had a total length of approximately 70 mm, a thickness of 22 mm in the gauge section, and a reduced-section diameter of 22.6 mm. The core sand mixtures were prepared using two phenol-formaldehyde resole-type resins differing in reactivity, together with an ester hardener added at 25 wt.% relative to the resin content. Two high-purity quartz sands supplied by DB Cargo—BK 40 and BK 45—were used as the base material; these sands differ in grain size distribution and specific surface area. BK 40 sand exhibits an average grain size of 0.30–0.35 mm (AFS ≈ 40), whereas the BK 45 fraction ranges from 0.25 to 0.30 mm. The SiO_2_ content in both sands exceeded 99.5%, and the grain size distributions were narrow, allowing for a detailed assessment of the influence of matrix granulometry on mechanical properties, permeability, and the thermal behaviour of the cores. The mixture composition corresponded to 100 parts by weight of sand, 1.5 parts by weight of resin, and 0.375 parts by weight of hardener. After thorough mixing, the sand mixtures were compacted using a LUZ-1 vibrational device (WADAP, Multiserw-Morek, Marcyporęba, Poland). To ensure comparability and repeatability, all experiments were performed under identical environmental conditions (T = 20 ± 1 °C, RH = 45–50%), using the same mixing procedure, curing time intervals, specimen geometry, and testing equipment. The reference system for comparative analysis consisted of core sands prepared with the same binder dosage and curing conditions, differing only in resin reactivity and sand granulometry. The applied testing procedures were based on commonly accepted foundry testing methodologies and recommendations described in the literature [2,4,18,29], including standardised approaches for tensile strength, friability, permeability, and thermal deformation measurements used in foundry practice.

The experimental programme included the following tests:

Tensile strength as a function of curing time ($R_{m}^{u}$) was determined using an LRu-2e universal strength testing machine (Multiserw-Morek, Marcyporęba, Poland), following a uniaxial tensile testing procedure. Each specimen was placed between the grips of the testing machine and subjected to an axially applied load that increased linearly until fracture. The maximum tensile stress was calculated based on the recorded load and specimen geometry. Measurements were conducted after 1, 3, 24, and 48 h from mixture preparation, enabling evaluation of curing kinetics and the development of binder mechanical strength over time.

Friability (S^u^) was determined using an HSW testing device (Huta Stalowa Wola, Stalowa Wola, Poland). A cured cylindrical specimen was fixed in a holder and rotated at a speed of one revolution per second, driven by a motor with a reduction gearbox. A total of 1750 g of steel shot with a diameter of 1 mm was used in the test. After completion, the specimen was reweighed with an accuracy of 0.1 g, and the friability value (S^u^) was calculated based on the mass loss before and after the test.

Permeability (P^u^) was measured using an LPiR-1 apparatus (Multiserw-Morek, Marcyporęba, Poland). During the test, the specimen was placed in the measuring chamber, through which an air stream at a pressure of approximately 980 Pa was passed. This method allows assessment of the ability of the porous core structure to transmit gases generated during pouring with molten metal.

Thermal deformation (Hot Distortion) tests were performed using a DMA device (Multiserw-Morek, Marcyporęba, Poland). The measurement involved recording changes in specimen shape as a function of temperature and time. Specimens with dimensions of 114 × 25.4 × 6.3 mm were heated from 25 °C to 350 °C at a heating rate of 10 °C·min^−1^. For each core sand composition, two independently prepared specimens were tested (n = 2). The reported results correspond to the mean values calculated from the two measurements. Repeatability of the results was evaluated based on independent specimen preparation and testing under identical experimental conditions.

#### 2.2.2. Thermophysical Properties

**Figure 2:**
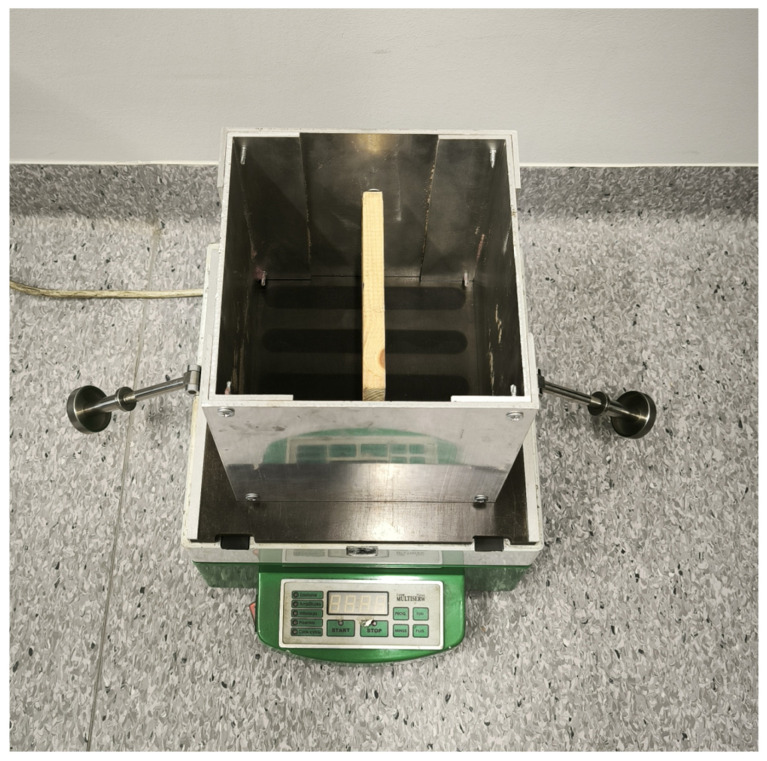
Experimental setup for the preparation of moulds used in thermophysical parameter measurements.

For the determination of thermophysical parameters, mixtures of quartz sand, resin, and hardener were prepared using the same component proportions and material parameters as those applied in the technological and mechanical tests. Four different moulding sand mixtures were produced, each with a mass of approximately 11 kg. The mixtures were compacted using a vibrational device in a moulding box with dimensions of 182 × 190 × 198 mm. The compaction setup, together with the moulding box and the pattern, is shown in Figure 2. The geometry of the plate casting corresponded to a pattern with dimensions of 150 × 150 × 15 mm.

After binder curing, the moulds were poured with technically pure copper. During solidification and subsequent cooling of the casting, temperature was recorded within the casting, at the casting-mould interface, and in the moulding sand at distances of 2 mm, 4 mm, 6 mm, 8 mm, 10 mm, 12 mm, and 14 mm from the casting surface. Cooling curves of the casting and heating curves of the moulding sand were obtained. The experimental data collected were used for further calculations of the thermophysical coefficients of the moulding sand. The Gaussian error function method was applied to describe the temperature field. The fundamental equation used in subsequent calculations is the error function *erf* (*u*), expressed as

(1) $$erf\left(u\right)=\frac{T_{x}-T_{surface}}{T_{ambient}-T_{surface}}$$

where *T_x_*—is the temperature measured at a distance x from the casting surface; *T_surface_*—is the initial temperature of the mould; *T_ambience_*—is the constant temperature at the casting-mould interface.

Knowing the value of the error function erf(u), the inverse function is used to determine the parameter u, which constitutes the fundamental quantity for calculating the thermal diffusivity coefficient. The parameter u is expressed by the following equation:

(2) $$u=\frac{x}{2\sqrt{a_{2}\tau }}$$

Accordingly, the following quantities can be directly calculated: thermal diffusivity coefficient, *a*_2_:

(3) $$a_{2}={\left(\frac{x}{2u\sqrt{\tau }}\right)}^{2}$$

Heat accumulation coefficient, *b*_2_:

(4) $$b_{2}=\sqrt{\lambda_{2}C_{2}\rho_{2}}$$

Thermal conductivity coefficient, *λ*_2_:

(5) $$\lambda_{2}=a_{2}\rho_{2}C_{2}$$

where *τ*—the time that has elapsed since the mould was completely filled. The thermal impact of the plate casting then works in accordance with the assumptions of the half-space. *ρ*_2_ is the density of the moulding sand, and *C*_2_—is the specific heat capacity of the moulding sand [29,30,31].

According to the nomenclature adopted by the authors (Lewandowski, Longa, Zych), subscript 1 refers to parameters associated with the casting, whereas subscript 2 denotes parameters related to the mould material. Based on Equations (1) and (3)–(5), together with data on time, the distance of the thermocouple from the casting surface, and the temperature recorded by that thermocouple, it is possible to determine the thermophysical parameters of the sand mould material and their variation as a function of temperature.

## 3. Results and Discussion

### 3.1. Tensile Strength as a Function of Curing Time

**Figure 3:**
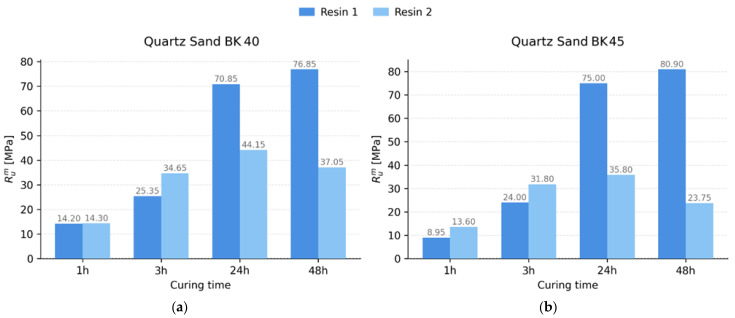
Tensile strength as a function of curing time for specimens made from core sand mixtures with Resin 1 and Resin 2: (**a**) mixtures based on quartz sand BK 40; (**b**) mixtures based on quartz sand BK 45.

The relationship between tensile strength and curing time is illustrated in Figure 3. At short curing times (1–3 h), higher tensile strength values were obtained for specimens prepared with the higher-reactivity resin (Resin 2), which is consistent with literature reports describing faster polycondensation stages of resoles with a higher degree of methylolation and a greater content of free -CH_2_OH groups [9,32]. At longer curing times (24–48 h), higher tensile strength was achieved by specimens containing the lower-reactivity resin (Resin 1). This behaviour can be explained by the fact that more reactive resoles (Resin 2) rapidly reach a high degree of conversion at an early stage of curing; however, further crosslinking may become limited. This limitation results from local stiffening of the network structure and reduced mobility of polymer segments, which hinders the formation of durable bonds in the later stages of curing [33]. Consequently, less reactive resins may exhibit a slower but more stable development of mechanical properties over time.

Sand granulometry also exerts a significant influence. The finer BK 45 fraction, in accordance with granulometric analyses reported, among others, by Hudák [34], is characterised by a higher specific surface area, which promotes more uniform grain coating and more effective formation of binder bridges. This effect is particularly evident for mixtures containing Resin 1, where the tensile strength values after 24 and 48 h are higher for BK 45 sand than for BK 40. This behaviour results from the increased total interfacial contact area between the organic and mineral phases, as previously confirmed for alkali-phenolic binder systems [4]. For compositions containing Resin 2, it is observed that at short curing times (1–3 h) specimens prepared with BK 45 sand exhibit higher tensile strength than those based on the BK 40 fraction. This may be attributed to the higher specific surface area of the finer sand, which facilitates faster formation of bonding bridges and accelerates the crosslinking process at the initial stage of the chemical reaction. However, after 48 h, a decrease in tensile strength relative to the BK 40 fraction is observed, which can be associated with the intense initial reaction and a possible limitation of polymer chain mobility at later stages of curing [32,35].

### 3.2. Friability

**Figure 4:**
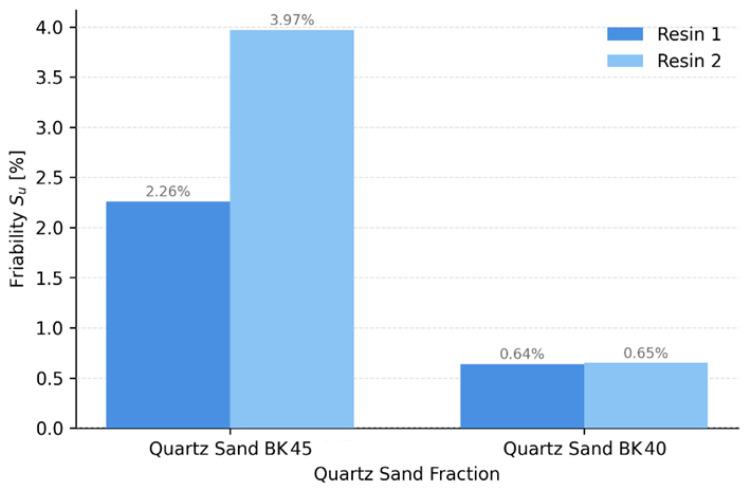
Friability of specimens made from core sand mixtures with Resin 1 and Resin 2.

The results presented in Figure 4 confirm that the surface friability of the cores depends on two main factors: (I) it increases with decreasing sand grain size and (II) it increases with increasing resin reactivity.

Based on the data shown in Figure 4, it can be concluded that core sands prepared with finer quartz sand (BK 45) exhibit a higher susceptibility to abrasion, particularly when combined with Resin 2. The rapid crosslinking rate of the more reactive resin may lead to pronounced differences in the degree of crosslinking (so-called conversion gradients), especially in the sand grain-binder interfacial region, where local stress concentrations may develop. This promotes the formation of a brittle near-surface layer, resulting in increased friability. This effect is clearly observed for the finer BK 45 sand, for which a mass loss of 3.97% was recorded for Resin 2, compared with 2.26% for BK 40 sand. This mechanism is consistent with observations reported for no-bake systems, where excessively rapid curing and/or unfavourable thermal conditions lead to the formation of friable surfaces with reduced mechanical resistance [36]. In contrast, the BK 40 fraction, characterised by a lower specific surface area, yielded significantly lower friability values (≈0.64–0.65%), regardless of the resin used. The observed reduction in friability associated with the use of a finer sand fraction, despite a lower overall binder content, may be interpreted as an effect of improved grain coating efficiency and denser packing of the sand matrix. The higher specific surface area of BK 45 sand enables a more uniform distribution of the resin on the grain surfaces, promoting the formation of more numerous and better-distributed polymer bridges. As a result, a more continuous and mechanically stable binder network is formed, reducing the number of potential initiation sites for mechanical damage in the near-surface layer. Such a microstructure may exhibit enhanced resistance to abrasion despite a lower total content of the organic phase [34,37].

### 3.3. Permeability

**Figure 5:**
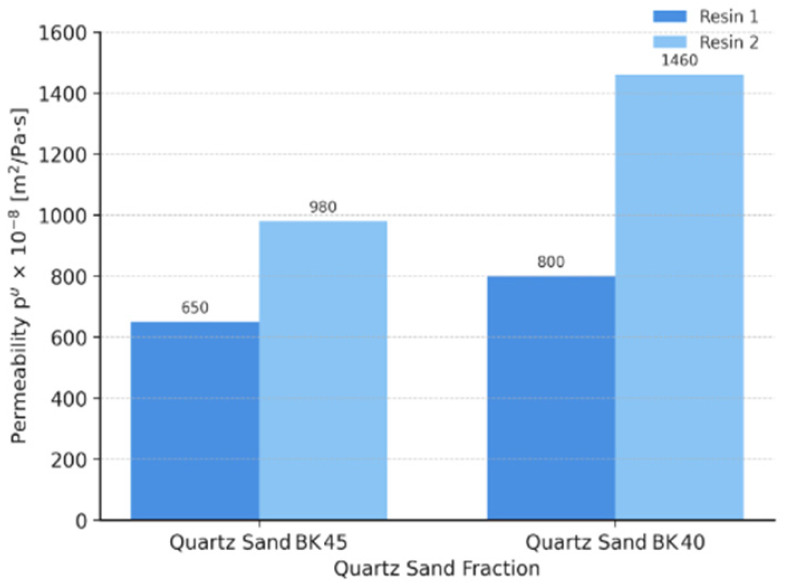
Permeability results for specimens made from core sand mixtures with Resin 1 and Resin 2.

The permeability results (Figure 5) show a clear increasing trend with increasing quartz sand grain size. This behaviour is consistent with expectations, as coarser grains are characterised by a lower specific surface area and lead to the formation of a more open sand matrix structure, resulting in a higher volume of interconnected flow pores within the sand-binder system.

For the BK 45 fraction, permeability values of 650 × 10^−8^ m^2^·Pa^−1^·s^−1^ (Resin 1) and 980 × 10^−8^ m^2^·Pa^−1^·s^−1^ (Resin 2) were recorded, whereas for the BK 40 fraction, the corresponding values were 800 × 10^−8^ m^2^·Pa^−1^·s^−1^ and 1460 × 10^−8^ m^2^·Pa^−1^·s^−1^, respectively. The increased permeability of cores produced with coarser sand is most likely attributable to the more open matrix structure and the greater number of flow pores, which facilitate the free transport of gases through the core. The higher permeability values observed for mixtures containing the more reactive resin may, in turn, be associated with differences in the crosslinking process. Faster curing promotes the formation of binder bridges with a less homogeneous structure and local concentrations of the organic phase. As a result, the polymer network may develop unevenly, leading to the formation of additional gas flow channels and an overall increase in core permeability. The observed differences in permeability are consistent with mechanisms described for no-bake systems, in which coarser sand fractions favour the formation of a more open matrix microstructure. In the analysed system, this effect may be further amplified by the crosslinking characteristics of the applied resins, highlighting the important role of synergy between the physical parameters of the sand matrix and the kinetics of the binder’s chemical reactions [37].

### 3.4. Thermal Deformation (DMA)

**Figure 6:**
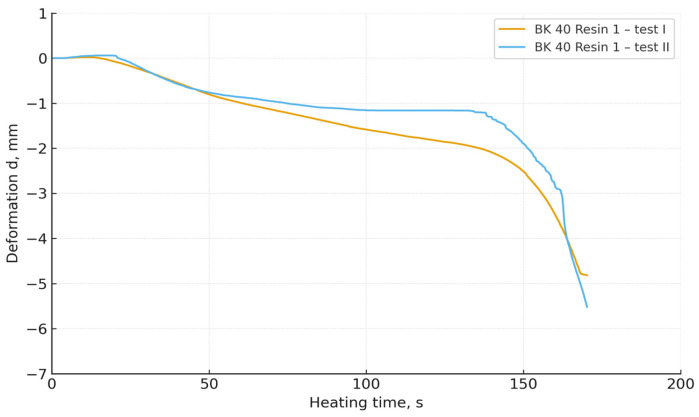
Thermal deformation of core sand specimens made with Resin 1 and BK 40 base sand.

**Figure 7:**
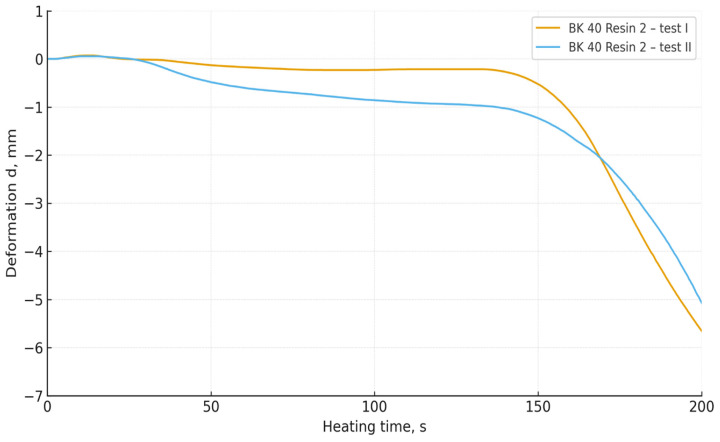
Thermal deformation of core sand specimens made with Resin 2 and BK 40 base sand.

**Figure 8:**
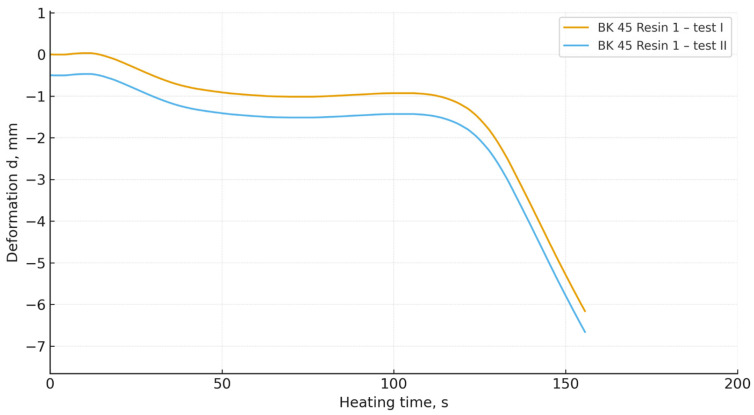
Thermal deformation of core sand specimens made with Resin 1 and BK 45 base sand.

**Figure 9:**
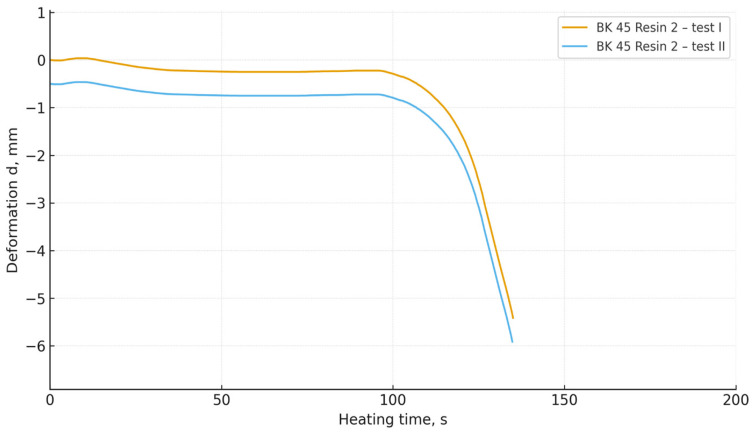
Thermal deformation of core sand specimens made with Resin 2 and BK 45 base sand.

The obtained DMA curves (Figure 6, Figure 7, Figure 8 and Figure 9) indicate that both the granulometry of the sand matrix and the reactivity of the applied phenol-formaldehyde binder have a significant influence on the thermomechanical resistance of the cores.

Foundry sand moulds prepared with BK 40 sand (the coarser fraction) exhibited a clearly more stable deformation behaviour during heating. In the initial heating stage, the specimens maintained dimensional stability, while a rapid decrease in deformation occurred only after prolonged thermal loading. Coarser grains are characterised by a lower specific surface area and a more open pore structure, which reduces the rate of heat transfer and delays the thermal degradation of resin bridges [37,38,39].

In contrast, core sands prepared with BK 45 sand (the finer fraction) exhibited an earlier loss of thermal stability and a more dynamic deformation response under heating. This behaviour can be attributed to the increased specific surface area of the finer grains, which results in a larger interfacial contact area between the organic and mineral phases, thereby intensifying local heating and accelerating the thermal degradation of the binder structure.

Regardless of the sand type used, mixtures containing Resin 2 (the higher-reactivity binder) showed a faster initiation of deformation. These results are consistent with the crosslinking mechanism of resole binders: more reactive resins form networks with a higher crosslink density, but are also more susceptible to rapid thermal degradation at elevated temperatures [33]. In contrast, mixtures containing Resin 1 (the lower-reactivity binder) exhibited slower deformation, indicating a more gradual degradation of polymer bridges and greater thermomechanical stability.

### 3.5. Determination of Thermophysical Coefficients

**Figure 10:**
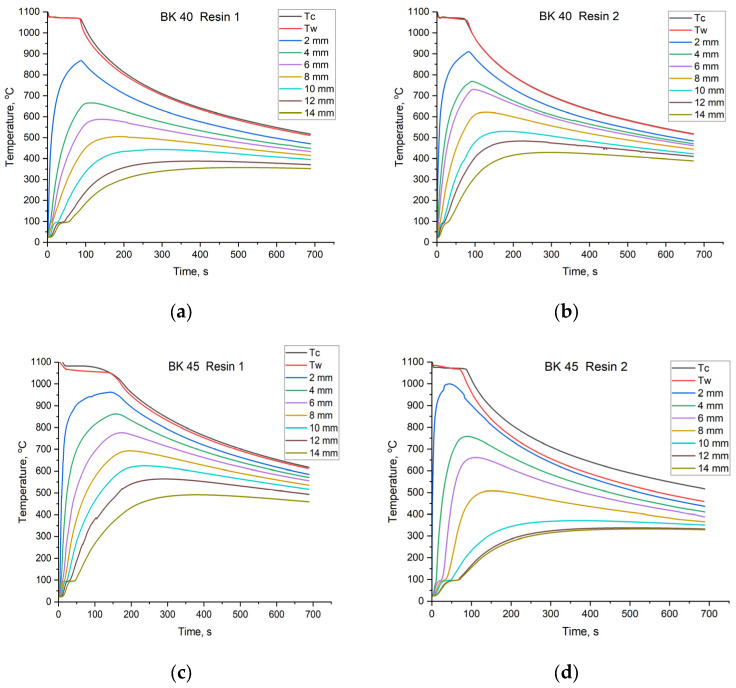
Cooling curves of the Cu plate casting and heating curves of the mould recorded during solidification and cooling at specified distances from the casting surface: (**a**) mixture of BK 40 quartz sand and Resin 1; (**b**) mixture of BK 40 quartz sand and Resin 2; (**c**) mixture of BK 45 quartz sand and Resin 1; (**d**) mixture of BK 45 quartz sand and Resin 2.

After binder curing, the prepared sand moulds were poured with technically pure copper at a temperature of approximately 1130 °C. During cooling of the molten metal, solidification of the casting, and subsequent cooling of the solidified casting, temperature was recorded both in the metal and in the mould. Figure 10 presents the cooling curves of the casting and the heating curves of the mould. The temperature $T_{c}$ corresponds to the thermocouple placed at the geometric centre of the casting. The temperature $T_{w}$ was recorded by a thermocouple positioned along the geometric axis of the plate casting at the casting-mould interface. The remaining thermocouples recorded temperature at specified distances from the casting surface.

The cooling curve of a casting made from technically pure copper exhibits a shape characteristic of the solidification and cooling of pure metals. After the initial cooling of the molten metal, crystals of the solid phase begin to form. From this moment, the release of latent heat of solidification occurs, resulting in a horizontal segment on the cooling curve. After complete solidification, the casting cools down to ambient temperature. In the analysed casting-sand mould system, the Cu plate acted as a heat source responsible for heating the mould over time. Recording the temperature increase in the sand mould during the solidification stage—when the surface temperature of the casting remains constant—provides stable conditions for collecting data required to calculate thermophysical coefficients. The mould heating curves recorded at distances of 2 mm, 4 mm, 6 mm, 8 mm, 10 mm, 12 mm, and 14 mm from the casting surface exhibit a characteristic curved shape. Immediately after pouring the sand mould with molten metal, thermal interaction begins, and heat penetrates into the mould, causing a rapid increase in temperature, which is clearly visible on the heating curves. At temperatures around 100 °C, a short plateau is observed on all heating curves. This effect is associated with the evaporation of residual moisture contained in the foundry sand molud. Foundry sand mould is a porous material capable of absorbing moisture from the environment [40,41]. For curves recorded at a distance of 2 mm from the Cu plate surface, this plateau is almost imperceptible due to the rapid temperature rise and the low residual moisture content of the mould. In contrast, the further the measurement point is from the heat source, the longer the thermal effect associated with water evaporation persists on the heating curve. This behaviour results from moisture transport into the deeper regions of the mould, which increases local moisture content and, consequently, the amount of heat required for evaporation. The influence of residual moisture is typically neglected in data obtained using other laboratory methods. Specimens tested under laboratory conditions are usually specially prepared and often dried. Moreover, specialised measurement techniques frequently require the use of vacuum, which further removes moisture from the tested material. While such approaches provide accurate material data, they do not reflect the actual behaviour of the material under real conditions, where it is exposed to humid air. As a porous material, the sand mould is sensitive to variations in ambient humidity and readily absorbs moisture, particularly in its surface layers. Therefore, the method based on recording the temperature field in the mould during casting solidification most accurately reflects the real operating conditions and parameters of foundry sand moulds. Analysis of the curves shows that the temperature within the mould increases until the end of the solidification period of the plate casting. With the cessation of heat supply from the latent heat of crystallisation, the casting begins to cool, accompanied by a decrease in the temperature of the sand mould. Heat is then transferred from the warmer regions of the mould to regions of lower temperature.

**Figure 11:**
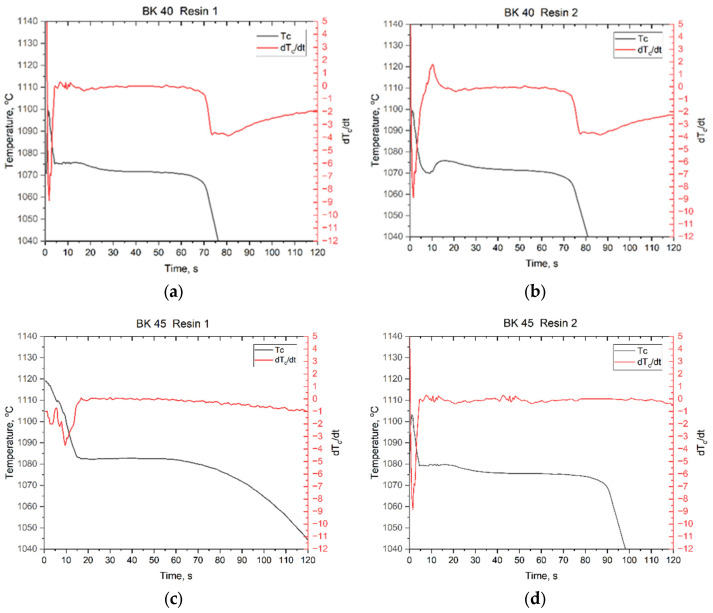
Solidification region of the Cu plate casting together with the first derivative of the temperature $T_{c}$ as a function of time t: (**a**) mixture of BK 40 quartz sand and Resin 1; (**b**) mixture of BK 40 quartz sand and Resin 2; (**c**) mixture of BK 45 quartz sand and Resin 1; (**d**) mixture of BK 45 quartz sand and Resin 2.

Figure 11 presents a fragment of the cooling curve of the plate casting in the solidification region together with the first derivative of the temperature $T_{c}$ with respect to time t. The plot of the first derivative allows the solidification time of the plate casting to be determined.

As can be seen from the plots, the solidification time varies with both the granulometry of the sand matrix and the type of binder used. The solidification time constitutes the first indication that the thermophysical parameters of the mould material differ depending on the composition of the foundry sand mould. For quartz sand BK 40 combined with Resin 1, the solidification time was 71 s, whereas for Resin 2 it was slightly longer, amounting to 73 s. The use of BK 45 quartz sand had a pronounced effect on the solidification time of the plate casting. When combined with Resin 1 as the binder, the mould removed heat more slowly, resulting in an extended solidification time of 89 s. For the combination of Resin 2 and BK 45 sand, the solidification time increased further to 92 s. It can be noted that the influence of the binder type on the solidification time is relatively minor, with differences on the order of 2–3 s. In contrast, the granulometry of the sand matrix exerts a much stronger effect, with differences of approximately 20 s observed between BK 40 and BK 45 sands. This behaviour is associated with the rate of heat extraction by the mould, heat transport into its deeper regions, and the amount of heat that the mould is capable of accumulating.

**Figure 12:**
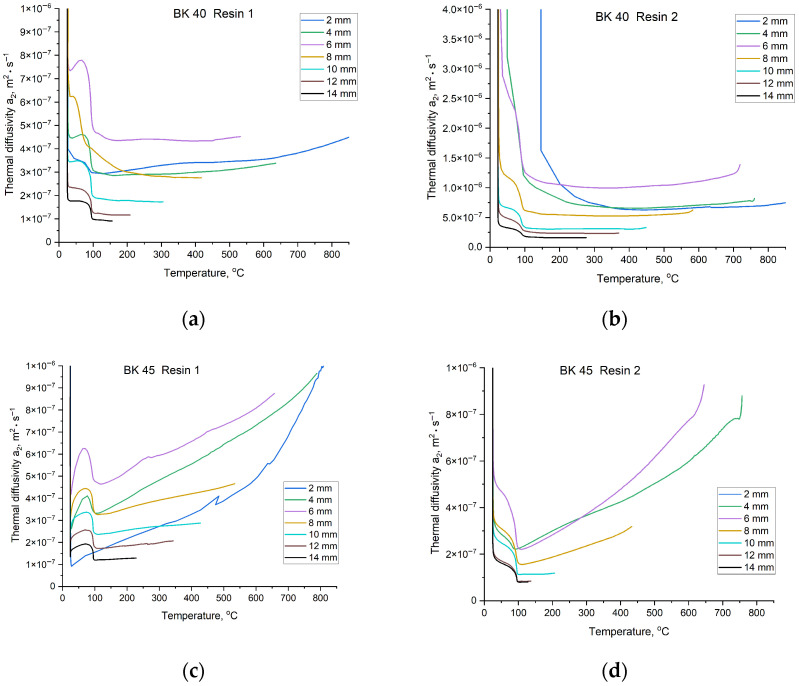
Values of the thermal diffusivity coefficient $a_{2}$ of the mould material for temperatures recorded at specified distances from the Cu plate casting surface: (**a**) mixture of BK 40 quartz sand and Resin 1; (**b**) mixture of BK 40 quartz sand and Resin 2; (**c**) mixture of BK 45 quartz sand and Resin 1; (**d**) mixture of BK 45 quartz sand and Resin 2.

Based on the cooling curves shown in Figure 10 and using Equations (1)–(3), the values of the thermal diffusivity coefficient $a_{2}$ were calculated as a function of temperature. The curves representing changes in $a_{2}$ with increasing sand mould temperature, depending on the location of the measurement point, are presented in Figure 12.

**Figure 13:**
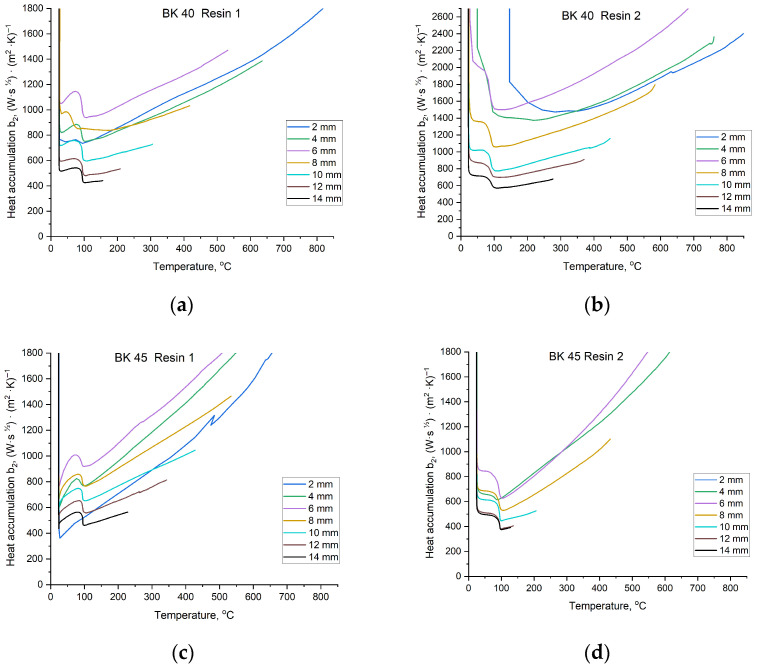
Values of the heat accumulation coefficient $b_{2}$ of the mould material for temperatures recorded at specified distances from the Cu plate casting surface: (**a**) mixture of BK 40 quartz sand and Resin 1; (**b**) mixture of BK 40 quartz sand and Resin 2; (**c**) mixture of BK 45 quartz sand and Resin 1; (**d**) mixture of BK 45 quartz sand and Resin 2.

All calculated thermophysical coefficients are interrelated and represent intrinsic properties of the investigated material. Using the calculated values of the thermal diffusivity coefficient $a_{2}$, the density of the sand mould, and Equation (4), the heat accumulation coefficient $b_{2}$ was determined. This coefficient defines the amount of heat that a given material used for foundry moulds is capable of storing. The values of the heat accumulation coefficient $b_{2}$ as a function of temperature and foundry sand mould composition are presented in Figure 13.

The investigated coefficients exhibit a strong dependence on temperature; therefore, knowledge of their temperature evolution is essential for accurately characterising mould material properties. The foundry mould exerts a significant influence on the properties of the casting produced within it. As shown in Figure 12 and Figure 13, the shapes of the curves vary depending on the mould material. The slope of each curve, reflecting changes in the coefficient value with time, is determined by the sensitivity of the parameter to temperature. When a curve exhibits a linear trend, it indicates that temperature changes do not significantly affect the parameter value, and the material displays more stable thermophysical properties.

**Figure 14:**
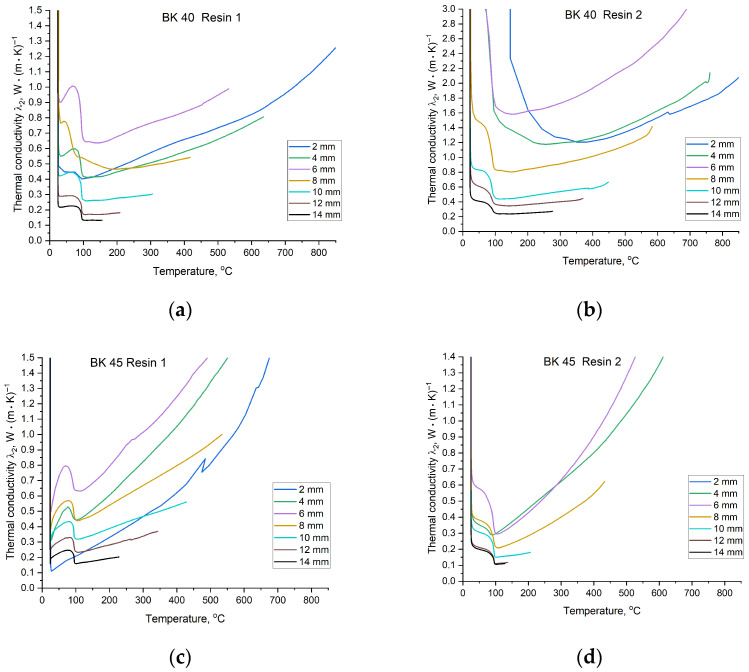
Values of the thermal conductivity coefficient $\lambda_{2}$ of the mould material for temperatures recorded at specified distances from the Cu plate casting surface: (**a**) mixture of BK 40 quartz sand and Resin 1; (**b**) mixture of BK 40 quartz sand and Resin 2; (**c**) mixture of BK 45 quartz sand and Resin 1; (**d**) mixture of BK 45 quartz sand and Resin 2.

The values of the thermal diffusivity coefficient $a_{2}$ and the heat accumulation coefficient $b_{2}$ can be divided into two temperature regions. The first extends from room temperature to approximately 100 °C, during which conditions stabilise after contact with molten metal, followed by a short equilibrium interval. Around 100 °C, a sharp decrease in coefficient values occurs due to intense evaporation of moisture from the mould. The final region, above approximately 100 °C, exhibits a more predictable, near-linear behaviour. Depending on the mould material, the coefficients vary to a greater or lesser extent with increasing temperature. A similar trend is observed for the last determined thermophysical parameter, namely the thermal conductivity coefficient $\lambda_{2}$. Changes in $\lambda_{2}$ values as a function of moulding sand composition are presented in Figure 14.

The thermal conductivity coefficient $\lambda_{2}$ characterises the rate of heat transfer into the interior of the mould. Analysis of the $\lambda_{2}$ trends indicates that moulds based on BK 40 sand exhibit greater stability of this parameter with increasing temperature. In contrast, the type of binder exerts a pronounced influence on $\lambda_{2}$ at specific measurement points. The application of Resin 2 in combination with BK 40 sand increased the value of $\lambda_{2}$ by approximately 0.9 W·m^−1^·K^−1^, thereby reducing the thermal insulation capability of the sand mould material. The same resin used with BK 45 sand did not produce comparable differences relative to Resin 1.

Observation of the $\lambda_{2}$ curves shows that, depending on the material configuration, the coefficient values either remain nearly constant or increase with temperature. For moulds prepared with BK 45 sand, a similar trend in $\lambda_{2}$ variation is observed regardless of the binder used, with a rapid increase in $\lambda_{2}$ as temperature rises. This behaviour is most likely related to the smaller diameter of the sand grains; although individual grains are finer, the total volume of intergranular voids is greater. Consequently, the moulding sand exhibits enhanced insulating properties, which is reflected in the observed changes in $\lambda_{2}$. This interpretation is consistent with the solidification time data: lower average values of $\lambda_{2}$ correspond to materials with higher thermal insulation capacity. In such materials, heat penetrates more slowly, delaying metal solidification and resulting in the extended solidification times observed in the cooling curves of the plate casting.

**Figure 15:**
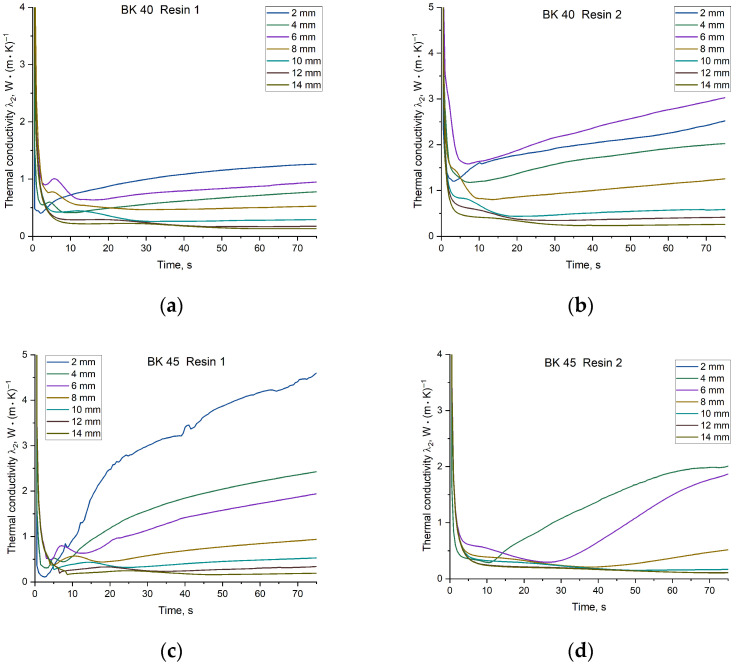
Values of the thermal conductivity coefficient $\lambda_{2}$ of the mould material as a function of solidification time for temperatures recorded at specified distances from the Cu plate casting surface: (**a**) mixture of BK 40 quartz sand and Resin 1; (**b**) mixture of BK 40 quartz sand and Resin 2; (**c**) mixture of BK 45 quartz sand and Resin 1; (**d**) mixture of BK 45 quartz sand and Resin 2.

Temperature-dependent changes in $\lambda_{2}$ provide important insight into mould material behaviour and its influence on the resulting casting microstructure. Of particular importance is the initial heating stage of the sand mould during casting solidification. A comparison of the temporal evolution of $\lambda_{2}$ during the solidification of the copper plate casting is presented in Figure 15. The value of $\lambda_{2}$ varies within the range of 5 to 0.1 W·m^−1^·K^−1^, with the most pronounced changes occurring during the evaporation of water contained in the mould. After this period, typically lasting approximately 10 s, the coefficient stabilises. Depending on the sand matrix and binder applied, $\lambda_{2}$ values measured at different distances from the casting surface converge to a greater or lesser extent. The most unstable measurements were obtained for thermocouples located closest to the solidifying casting, which is attributed to rapid changes in thermal conditions in this region of the mould. Due to the high temperatures prevailing in this zone, binder degradation occurs almost immediately after pouring with molten metal, leading to changes in the thermophysical parameters of the mould material.

## 4. Conclusions

Based on the conducted investigations of mechanical and technological properties, thermal deformation behaviour, and thermophysical parameters of core sands bonded with phenol-formaldehyde resole resins of different reactivity and prepared with two quartz sand fractions (BK 40 and BK 45), the following conclusions can be drawn:

(1) At short curing times (1–3 h), mixtures containing the more reactive resin achieved higher tensile strength values (≈0.42–0.45 MPa), whereas after 24–48 h, higher tensile strength was obtained for mixtures bonded with the less reactive resin (≈0.58–0.62 MPa), confirming a more stable crosslinking process for the latter.

(2) Higher resin reactivity (Resin 2) promoted increased core friability, which was particularly pronounced when combined with the finer BK 45 sand fraction. Although this configuration enabled higher strength levels to be achieved (e.g., ≈0.62 MPa for Resin 1 after 48 h), it simultaneously resulted in increased susceptibility to thermal degradation and higher friability.

(3) The highest friability was recorded for the BK 45 + higher-reactivity resin system (≈3.97%), while the lowest values were observed for BK 40 (≈0.64–0.65%), confirming greater surface stability for coarser grains. BK 40 sand provided the highest permeability (800–1460 × 10^−8^ m^2^·Pa^−1^·s^−1^), whereas BK 45 reduced permeability by 30–40%, increasing the risk of gas-related defects under industrial conditions.

(4) Mixtures based on BK 40 exhibited delayed deformation initiation and greater thermal stability, whereas BK 45 led to faster degradation of resin bridges and more dynamic deformation behaviour. Mixtures containing the more reactive resin deformed more rapidly and more intensively than those bonded with the less reactive resin.

(5) Core sands based on BK 40 were characterised by a more stable average thermal conductivity coefficient (*λ*_2_ ≈ 0.53–0.94 W·m^−1^·K^−1^) and higher thermal diffusivity (*a*_2_ ≈ 3.0–5.9 × 10^−7^ m^2^·s^−1^). However, for this sand fraction, the type of resin applied had a significant effect: Resin 2 increased the heat conduction capacity of the mould by approximately 0.8 W·m^−1^·K^−1^. In contrast, BK 45 sand exhibited a more insulating behaviour, resulting in higher heat accumulation (*b*_2_ up to ≈ 1282 W·s^1/2^/m^2^·K) and slower heat dissipation.

(6) Sand granulometry had a pronounced influence on the actual solidification time of the casting. Replacing BK 40 with BK 45 extended the solidification time of the Cu plate from 71–73 s to 89–92 s, corresponding to an increase of approximately 20 s and confirming the higher insulating capacity of the finer sand. Differences in solidification time attributable to the binder type did not exceed 2–3 s.

(7) From an industrial perspective, the BK 40 + lower-reactivity resin system provided the most balanced performance, combining high dimensional stability, low friability, higher permeability, and predictable thermal behaviour. The BK 45 + higher-reactivity resin configuration ensured rapid strength development but required more stringent process control (e.g., coatings, dosage adjustments, and stable storage conditions). The obtained temperature-dependent thermophysical coefficients can be directly implemented in foundry simulations, improving the accuracy of real-process representation and enabling informed selection of materials for moulds and cores.

Limitations of the present study include the investigation of a single binder system (phenol-formaldehyde resole) and two quartz sand fractions under controlled laboratory conditions. The analysis focused on selected curing times and did not consider long-term storage effects, cyclic thermal loading, or the influence of surface coatings applied to cores. In addition, the thermophysical parameters were determined for a specific casting geometry and alloy, which may influence the absolute values obtained. Future research will therefore focus on extending the approach to other binder systems, including inorganic and hybrid binders, as well as on the evaluation of long-term storage and ageing effects. Further studies will also address different casting geometries, alloys, and boundary conditions, and will integrate the experimentally determined temperature-dependent thermophysical coefficients into advanced numerical simulations of real casting processes.

## Figures and Tables

**Table 1:** Physicochemical characteristics of the alkaline resole resins and the hardener used in foundry core technology [17].

Property	Resin 1	Resin 2	Hardener
**Chemical type**	Alkaline phenolic (resole) resin	Alkaline phenolic (resole) resin	Mixture of dicarboxylic acid esters
**Reactivity**	Lower	Higher	Not applicable
**Solid content [%]**	24–26	48–52	—
**Alkalinity**	5–6% KOH + 3–4% NaOH	10–12% KOH	—
**pH (20 °C)**	11–13	12–14	—
**Viscosity (20 °C)**	120–260 mPa·s	130–170 mPa·s	—
**Density [g/cm^3^]**	1.20–1.25	1.22–1.26	1.076–1.096
**Residual phenol [%]**	≤0.23	≤0.4	—
**Free formaldehyde [%]**	<0.08	<0.10	—

## Data Availability

The original contributions presented in this study are included in the article material. Further inquiries can be directed to the corresponding author.

## References

[1] Czerwińska K., Pacana A., Ostasz G. (2025). A Model for Sustainable Quality Control Improvement in the Foundry Industry Using Key Performance Indicators. Sustainability.

[2] Sobczak J.J. (2013). Odlewnictwo Współczesne. Poradnik Odlewnika Tom 1: Materiały.

[3] Holtzer M., Kmita A. (2020). Mold and Core Sands in Metalcasting: Chemistry and Ecology.

[4] Łucarz M., Drożyński D., Jezierski J., Kaczmarczyk A. (2019). Comparison of the Properties of Alkali-Phenolic Binder in Terms of Selection of Molding Sand for Steel Castings. Materials.

[5] Obzina T., Merta V., Folta M., Bradáč J., Beňo J., Novohradská N., Gawronová M., Kroupová I., Lichý P., Radkovský F. (2021). Technological and Quality Aspects of the Use of Innovative Inorganic Binders in the Production of Castings. Metals.

[6] Fortini A., Merlin M., Raminella G. (2022). A Comparative Analysis on Organic and Inorganic Core Binders for a Gravity Diecasting Al Alloy Component. Int. J. Met..

[7] Kmita A. (2025). Phenolic Binders Based on Resole Resins for the Foundry Industry—Thermal Characteristics. Arch. Foundry Eng..

[8] Hu H., Wang W., Jiang L., Liu L., Zhang Y., Yang Y., Wang J. (2022). Curing Mechanism of Resole Phenolic Resin Based on Variable Temperature FTIR Spectra and Thermogravimetry–Mass Spectrometry. Polym. Polym. Compos..

[9] Thébault M., Kandelbauer A., Zikulnig-Rusch E., Putz R., Jury S., Eicher I. (2018). Impact of Phenolic Resin Preparation on Its Properties and Its Penetration Behavior in Kraft Paper. Eur. Polym. J..

[10] Santos C., Santos T., Fonseca R., Melo K., Aquino M. (2021). Phenolic Resin and Its Derivatives. Phenolic Polymers Based Composite Materials.

[11] Zhao W., Hsu S.L., Ravichandran S., Bonner A.M. (2019). Moisture Effects on the Physical Properties of Cross-Linked Phenolic Resins. Macromolecules.

[12] Woźniak F., Bobrowski A. (2025). Effect of Resin Reactivity and Storage Conditions on the Properties of Foundry Cores Based on Phenolic Resole Binders. Appl. Sci..

[13] Lu X., Han J., Shephard N., Rhodes S., Martin A.D., Li D., Xue G., Chen Z. (2009). Phenolic Resin Surface Restructuring upon Exposure to Humid Air: A Sum Frequency Generation Vibrational Spectroscopic Study. J. Phys. Chem. B.

[14] Strzemiecka B., Zieba-Palus J., Voelkel A., Lachowicz T., Socha E. (2016). Examination of the Chemical Changes in Cured Phenol-Formaldehyde Resins during Storage. J. Chromatogr. A.

[15] Bérot O.S., Hassoune-Rhabbour B., Acheritobehere H., Laforet C., Canot H., Durand P., Nassiet V. (2025). Study of the Hygrothermal Aging of a Phenolic Matrix and Glass Fiber Composite. J. Compos. Mater..

[16] Chiantore O., Lazzari M., Fontana M. (1995). Thermal Decomposition of Phenol-Formaldehyde Foundry Resins. Int. J. Polym. Anal. Charact..

[17] Bobrowski A., Woźniak F., Żymankowska-Kumon S., Ziętal H., Januszek K., Grabowska B. (2025). The Impact of Storage Conditions on the Gas-Forming Tendency of Moulds and Cores Made with Resole-Type Phenol Formaldehyde Resin. Materials.

[18] Zych J., Mocek J., Snopkiewicz T., Jamrozowicz M. (2015). Thermal Conductivity of Moulding Sand with Chemical Binders: Attempts of Its Increasing. Arch. Metall. Mater..

[19] Saeidpour M., Svenningsson R., Gotthardsson U., Farre S. (2021). Thermal Properties of 3D-Printed Sand Molds. Int. J. Met..

[20] Parker W.J., Jenkins R.J., Butler C.P., Abbott G.L. (1961). Flash Method of Determining Thermal Diffusivity, Heat Capacity, and Thermal Conductivity. J. Appl. Phys..

[21] Tye R.P. (1969). Thermal Conductivity.

[22] He Y. (2005). Rapid Thermal Conductivity Measurement with a Hot Disk Sensor: Part 1. Theoretical Considerations. Thermochim. Acta.

[23] Nagai H., Rossignol F., Nakata Y., Tsurue T., Suzuki M., Okutani T. (2000). Thermal Conductivity Measurement of Liquid Materials by a Hot-Disk Method in Short-Duration Microgravity Environments. Mater. Sci. Eng. A.

[24] Krajewski P.K., Piwowarski G., Buraś J. (2016). Thermal Properties of Foundry Mould Made of Used Green Sand. Arch. Foundry Eng..

[25] Jacquet P., Vaucheret A., Souêtre M., Carton J.F. (2024). Determination of Thermal Properties of Foundry Green Sand to Improve Numerical Simulation. Int. J. Met..

[26] Hou J., Le Q., Chen L., Jia Y., Hu C., Ben Seghier M.E.A. (2023). Domination and Effect of Multi-Parameters in Direct Chill Casting Based on Establishment of Thermo Model by Numerical Simulation and Experiment. J. Mater. Res. Technol..

[27] Chebykin D., Ohta H., Endo R., Volkova O. (2025). Methods for Thermal Conductivity and Thermal Diffusivity Measurements of Solid and Molten Mold Fluxes. Steel Res. Int..

[28] Przyszlak N., Piwowarski G. (2023). Designing of X46Cr13 Steel Heat Treatment in Condition of Casting Mould. Arch. Foundry Eng..

[29] Łągiewka M., Konopka Z., Zyska A., Nadolski M. (2013). Determination of Heat Accumulation Coefficient for Oil Bonded Moulding Sands. Arch. Foundry Eng..

[30] Krajewski W.K., Suchy J.S. (2010). Determining Thermal Properties of Insulating Sleeves. Mater. Sci. Forum.

[31] Longa W. (1985). Krzepnięcie Odlewów.

[32] Domínguez J.C., Alonso M.V., Oliet M., Rodríguez F. (2010). Chemorheological Study of the Curing Kinetics of a Phenolic Resol Resin Gelled. Eur. Polym. J..

[33] Gardziella A., Pilato L.A., Knop A. (2000). Phenolic Resins: Chemistry, Reactions, Mechanism. Phenolic Resins.

[34] Budavári I., Hudák H., Fegyverneki G. (2023). The Role of Acid Hardener on the Hardening Characteristics, Collapsibility Performance, and Benchlife of the Warm-Box Sand Cores. Arch. Foundry Eng..

[35] Kamarudin N., Biak D.R.A., Abidin Z.Z., Cardona F., Sapuan S.M. (2020). Rheological Study of Phenol Formaldehyde Resole Resin Synthesized for Laminate Application. Materials.

[36] Bobrowski A., Grabowska B. (2012). The Impact of Temperature on Furan Resin and Binder Structure. Metall. Foundry Eng..

[37] Hudák H., Gyarmati G., Varga L. (2021). Investigation on the Effect of Granulometric Features on the Permeability of No-Bake Resin Bonded Sand Cores. Arch. Foundry Eng..

[38] Ajay R., Lovneesh S. (2023). Experimental Study on Effect of Sand Grain Size and Heat Dissipation on the Properties of Moulding Sand. IOP Conf. Ser. Earth Environ. Sci..

[39] Midttømme K., Roaldset E. (1998). The Effect of Grain Size on Thermal Conductivity of Quartz Sands and Silts. Pet. Geosci..

[40] Zych J., Kaźnica N. (2015). Procesy pochłaniania wilgoci z otoczenia i wysychania na przykładzie wierzchnich warstw form odlewniczych—Moisture Sorption and Desorption Processes on the Example of Moulding Sands’ Surface Layers. Arch. Foundry Eng..

[41] Kaźnica N., Zych J. (2015). Investigations of the Sorption Process’ Kinetics of Sand Moulds’ Surface Layers under Conditions of High Air Humidity. Arch. Foundry Eng..

